# 肺腺癌组织中OCT4表达与患者临床指标及预后的相关性

**DOI:** 10.3779/j.issn.1009-3419.2013.04.05

**Published:** 2013-04-20

**Authors:** 雪艳 张, 慧敏 王, 波 金, 强刚 董, 进肃 黄, 宝惠 韩

**Affiliations:** 1 200030 上海，上海交通大学胸科医院呼吸内科 Department of Pulmonary Medicine, Shanghai Chest Hospital, Shanghai Jiaotong University, Shanghai 200030, China; 2 200030 上海，上海交通大学肿瘤研究所 Cancer Stem Cells Section, Shanghai Cancer Institute, Shanghai Jiaotong University, Shanghai 200030, China

**Keywords:** 肺肿瘤, OCT4, 预后, Lung neoplasms, OCT4, Prognosis

## Abstract

**背景与目的:**

肺腺癌发病率不断升高, 而OCT4是调控干细胞自我更新的关键基因, 在癌干细胞的增殖分化过程中起重要调节作用。本研究目的为检测肺腺癌组织中OCT4表达, 并分析其与肺腺癌患者转移、化疗疗效及预后的相关性。

**方法:**

采用免疫荧光方法检测肺腺癌组织OCT4表达。卡方检验OCT4表达与临床病理指标的关系, *Kaplan-Meier*生存曲线计算生存率, 采用*Cox*分析评估各指标与患者生存之间的关系。

**结果:**

126例肺腺癌组织中, 91例可观察到OCT4阳性细胞。OCT4表达与肺腺癌患者转移及化疗耐药密切相关, 且与患者无病生存期和生存期呈明显负相关。

**结论:**

OCT4表达与肺腺癌患者的转移及化疗耐药相关, 提示预后不良。

## Abstract (en)

**Background and objective:**

In recent years, cases of lung adenocarcinoma morbidity have consistently grown.OCT4 is the key gene that controls the automatic renewal of stem cells, and regulates the proliferation and differentiation of cancer stem cells.The aim of this study is to detect OCT4 expression in lung adenocarcinoma tissues, and to evaluate its relevance in the metastasis, chemotherapeutic effect, and prognosis in lung adenocarcinoma patients.

**Methods:**

Immunofluorescence method was employed to detect OCT4 expression in lung adenocarcinoma tissues.The relationship between OCT4 expression and clinical pathological indicators is examined through chi-square test.Moreover, the survival rate is calculated through the *Kaplan-Meier* survivorship curve.Finally, the relevance between the indicators and patient survival is estimated using *Cox* analysis.

**Results:**

Among the 126 tissue samples of lung adenocarcinoma, 91 showed OCT4 positive cells.OCT4 expression is closely related to metastasis and chemoresistance in lung adenocarcinoma patients, and negatively corresponds to the patients' disease-free survival and survival periods.

**Conclusion:**

OCT4 expression is related to metastasis and chemoresistance in lung adenocarcinoma patients, and thus indicates poor prognosis.

癌干细胞(cancer stem cells, CSC)被认为是恶性肿瘤的起始细胞, 能通过自我更新获得无限增殖能力, 并可分化形成肿瘤内各种癌细胞类型, 鉴于此, CSC作为肿瘤诊治的重要靶标已日益引起业界的高度关注。

肺腺癌是肺癌的常见组织类型之一, 恶性程度高, 预后差, 且发病率近年来有明显上升趋势, 因而对其细胞起源及癌变机制的研究一直是肺癌治疗的主攻方向之一。近年来关于胚胎干细胞(embryonic stem cells, ESC)自我更新和全能分化机理的研究已取得明显进展。资料显示, ESC自我更新主要在基因转录水平受到精细调控, 如OCT4被公认是ESC的核心调控基因。根据目前对干细胞自我更新机制的认识, OCT4在干细胞控制自我更新、增殖分化及致瘤转移中起重要作用。本研究拟通过检测肺腺癌组织中干细胞标志OCT4的表达, 分析其与临床指标的关系及对患者预后的影响。

## 1 资料与方法

### 1.1 临床资料

收集上海交通大学附属胸科医院1999年1月-2004年6月经外科根治性手术切除且随访资料完整的126例肺腺癌患者进入本研究。所有患者术前均未进行过放疗或化疗, 术后接受2次-4次以铂类为主的化疗, 本组病例均具有完整的随访资料。患者年龄22岁-80岁, 中位年龄57岁; 男性65例, 女性61例。按国际抗癌联盟标准进行分期^[1]^, ⅠB期30例, Ⅱ期39例, Ⅲa期57例。高分化30例, 中分化76例, 低分化20例。随访从手术之日开始, 进行电话随访, 末次随访日为2009年12月15日, 本组患者的随访时间均 > 5年。随访内容主要为:是否生存、死亡原因、死亡时间、转移部位、转移时间、治疗方案等。手术标本经4%甲醛固定, 石蜡包埋, 制成4 μm厚切片。由两位病理科主任医师对原发病灶的病理切片进行审核, 明确病理诊断。

### 1.2 免疫荧光检测

取切除的肺腺癌组织的石蜡切片, 经脱蜡及抗原修复后, 采用间接法进行免疫荧光染色。具体操作步骤如下:脱蜡及抗原修复后的石蜡切片放入5%脱脂奶粉溶液(5 g脱脂奶粉:100 mL PBS)中室温封闭1 h, 经pH 7.3的PBS冲洗后, 加鼠抗人OCT4单克隆抗体100 μL(Santa Cruz公司, sc-5279, 1:50稀释, 即在98 μL PBS中加一抗2 μL), 在对照的组织切片上只加100 mL PBS。4 ℃温育过夜。PBS冲洗3次后, 分别用罗丹明标记及荧光素异硫氰酸酯(FITC)标记的驴抗鼠多克隆抗体100 μL(Santa Cruz公司, 1:100稀释, 即在99 μL PBS中加二抗1 μL)室温染色2 h, 染色后切片经PBS冲洗3次, 每次5 min, 加上100 μL Hoechest33342(1:50稀释)染核, 常温避光4 min并封片。封片后在荧光显微镜下观察。

### 1.3 免疫荧光评定标准

OCT4阳性表达细胞为胞浆染色, 呈绿色荧光, 核呈蓝色荧光(图 1)。

**1:**
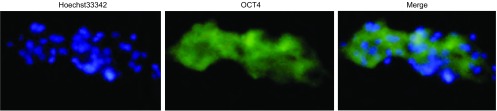
肺腺癌组织中OCT4表达的免疫荧光检测结果 Immunofluorescence staining of OCT4 in lung adenocarcinoma

### 1.4 统计学方法

实验数据应用SPSS 11.0统计软件进行分析处理。组间率的比较采用*χ*^2^检验。*Kaplan-Meier*方法计算生存曲线, 生存分析采用*Cox*多因素分析。以*P* < 0.05为差异有统计学意义。

## 2 结果

### 2.1 OCT4在肺腺癌组织中的表达

126例肺腺癌组织中91例表达OCT4, 呈绿色荧光。35例表达为阴性。免疫荧光检测见图 1。

### 2.2 OCT4表达与临床病理特征的关系

表 1显示, OCT4表达与患者年龄、性别、吸烟史、肿块大小、肿瘤分化、病理分期等指标均无明显相关性(*P* > 0.05)。

**1:** OCT4表达与肺腺癌临床病理特征的相关性 Correlation between OCT4 and clinicopathological factors in 126 patients with lung adenocarcinoma

Characteristics	*n*	OCT4		*χ*^2^	*P*
		-	+		
Age (yr)				0.658	0.404
< 65	83	22	61		
≥65	43	13	30		
Gender				0.707	0.430
Male	65	19	46		
Female	61	16	45		
Smoking status				0.223	0.158
< 400	88	28	60		
≥400	38	7	31		
Tumor size				0.927	0.542
≤3 cm	46	13	33		
> 3 cm	80	22	58		
Pathological stages				0.956	0.620
Ⅰb	30	7	23		
Ⅱ	39	13	26		
Ⅲa	57	15	42		
T stage				0.435	0.177
1	8	3	5		
2	97	30	67		
3	21	2	19		
N stage				2.251	0.325
0	33	7	26		
1	45	16	29		
2	48	12	36		
Differentiation				1.866	0.393
Well	30	7	23		
Moderate	76	20	56		
Poor	20	8	12		
Statistical analyses were performed using Pearson Chi-square test.^*^P < 0.05.					

### 2.3 OCT4表达与转移和疗效的关系

126例患者中, 95例发生转移, 其中78例为OCT4阳性表达。由于转移后部分患者未进行化疗, 仅85例患者可随访到转移后化疗疗效。表 2显示, OCT4表达与转移密切相关, 且与转移后化疗疗效明显呈负相关。而与转移途径及转移部位无相关。

**2:** OCT4表达与肺腺癌患者的转移及疗效的相关性 Correlation between OCT4 and metastasis and chemotherapy efficacy in 126 patients with lung adenocarcinoma

Characteristic	*n*	OCT4		*χ*^2^	*P*
		-	+		
Metastasis	126				
Yes	95	17	78	18.799	< 0.001^﹡^
No	31	18	13		
Metastasis pathway	95				
Lymph	20	3	17	0.144	0.496
Blood	75	14	61		
Metastasis site	95				
Lung	31	3	28	6.760	0.239
Brain	15	2	13		
Bone	20	5	15		
Other parts	9	4	5		
Mediastinal lymphnode	13	2	11		
Supraclavicular lymphnode	7	1	6		
Chemotherapy efficacy	85				
PR	4	3	1	12.718	0.002^﹡^
SD	47	10	37		
PD	34	2	32		
Statistical analyses were performed using Pearson Chi-square test.^*^P < 0.05.PR:partial response; SD:stable disease; PD:progressive disease.					

### 2.4 OCT4表达与肺腺癌患者无病生存期及生存期的相关性

用*Log-rank*及生存曲线进行分析, 结果表明, OCT4表达与无病生存期明显相关(*P* < 0.001)(图 2)。图 3则显示OCT4表达对患者生存期的影响, OCT4阳性表达的患者较阴性表达的患者生存期明显缩短, 预后差(*P*=0.004)(图 3)。

**2:**
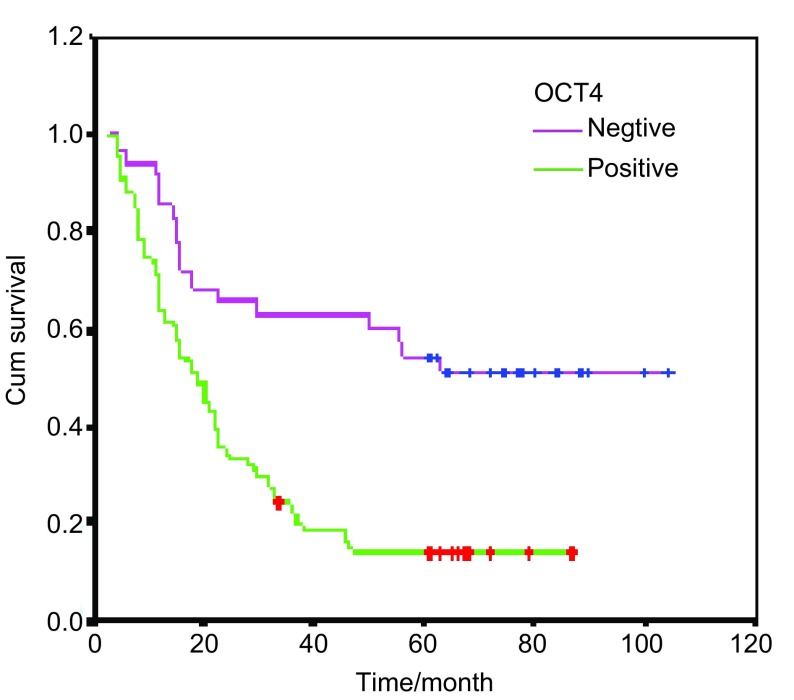
OCT4与无病生存期的相关性 Cumulative *Kaplan-Meier* survival curves for disease free time

**3:**
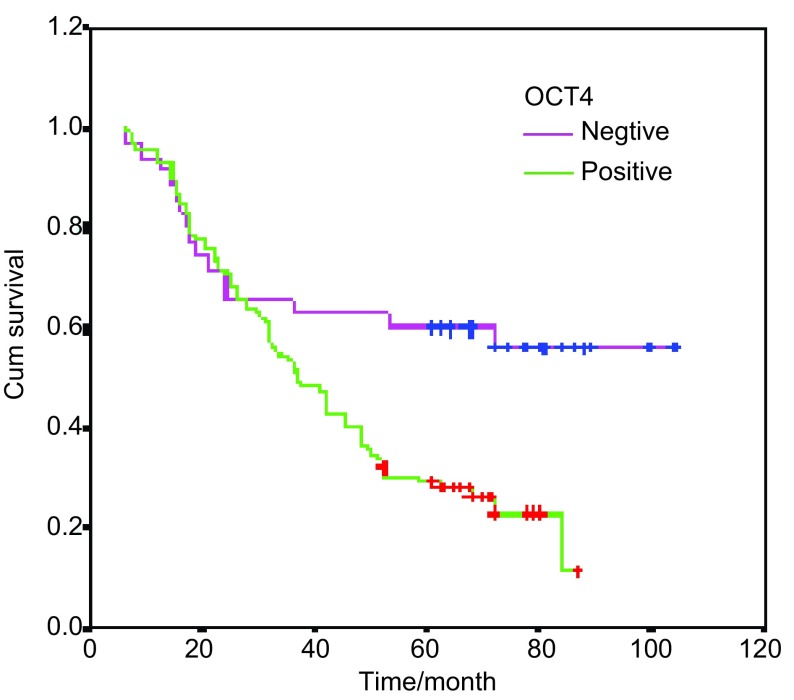
OCT4与生存期的相关性 Cumulative *Kaplan-Meier* survival curves for survival

### 2.5 肺腺癌患者生存期影响因素的*Cox*回归模型分析

单因素分析表明:年龄、病理分期、N分期、是否转移、转移后化疗疗效、OCT4表达均影响患者的生存。通过多因素分析显示转移、OCT4表达及转移后化疗疗效均是影响生存期的独立因素。OCT4表达阳性的患者生存期短(*P*=0.007, R=2.455, 95%CI:1.280-4.709)。此外, 转移后化疗效果好的患者也比化疗效果不佳的患者预后好(*P* < 0.001, R=2.380, 95%CI:1.680-3.372)。而转移也是预后的独立影响因素(*P* < 0.001, R=0.002, 95%CI:0.000-0.019)(表 3)。

**3:** 肺腺癌患者生存期影响因素的*Cox*回归模型分析结果 Univariate and multivariate analysis of clinicopathological factors for the overall survival rate of 126 patients with lung adenocarcinoma

Parameter	*β*	S.E.	Wald	Exp(B)	95%CI for Exp(B)		*P*
					Lower Upper		
Univariate analysis							
Gender	0.024	0.220	0.012	1.025	0.666	1.577	0.912
Age	0.481	0.225	4.572	1.618	1.041	2.516	0.032^*^
Smoking status	0.104	0.220	0.222	1.109	0.721	1.708	0.637
Tumor size	-0.014	0.229	0.004	0.986	0.630	1.544	0.952
Pathological stage	0.346	0.145	5.673	1.414	1.063	1.879	0.017^*^
T stage	0.077	0.183	0.176	1.080	0.754	1.547	0.675
N stage	0.297	1.141	4.430	1.345	1.021	1.773	0.035
Differentiation	0.086	0.189	0.209	1.090	0.753	1.578	0.647
Metastasis	-4.210	1.014	17.236	0.015	0.002	0.108	< 0.001
Chemotherapy efficacy	-0.274	0.113	5.841	0.760	0.609	0.949	0.016
OCT4	-0.815	0.291	7.813	0.443	0.250	0.784	0.005
Multivariate analysis							
Metastasis	-6.212	1.150	29.201	0.002	0.000	0.019	< 0.001
OCT4	0.898	0.332	7.307	2.455	1.280	4.709	0.007
Chemotherapy efficacy	0.867	0.178	23.791	2.380	1.680	3.372	< 0.001
Pathological stage	0.495	0.336	2.167	1.640	0.849	3.169	0.141
N stage	-0.623	0.333	3.502	0.536	0.279	1.030	0.061
Age	0.195	0.256	0.581	0.215	0.736	2.005	0.446

## 3 讨论

按肿瘤的干细胞起源理论, 恶性肿瘤中存在一群数量极少的肿瘤干细胞, 这群细胞具有无限增殖、自我更新及分化的能力, 在启动肿瘤形成和生长中起着决定性的作用。目前研究^[2, 3]^认为, 肺癌中的肿瘤干细胞是肺癌复发转移及耐药的根源。

近年来, 关于ESC自我更新机制的研究^[4]^结果显示, OCT4与Nanog和Sox2转录因子形成核心调节环, 通过16种主调节基因来控制2, 260种mRNA转录, 后者构成了ESC特异性功能基因组。选择性敲除上述核心调节环的任何一个基因, 均可导致ESC失去自我更新能力而分化^[5, 6]^。因此, OCT4在ESC维持自我更新和全能性分化中起关键作用。

业已证明, CSC与ESC一样, 其自我更新功能亦受OCT4的调控。研究^[7]^显示在恶性肿瘤中也存在OCT4的表达, 并将这种现象归结为恶性肿瘤引起ESC未分化基因的再表达。近年来发现多种人体肿瘤(如乳腺癌、精原细胞瘤和肝癌等)的CSC表达OCT4^[8-10]^, 但在肺癌中报道不多^[11]^。本研究发现肺腺癌组织中表达OCT4, 提示肺腺癌中可能存在具有自我更新和分化潜能的干细胞样细胞。而OCT4表达与患者年龄、性别、吸烟史、肿块大小、病理分期、T分期、N分期及肿瘤分化等指标无明显相关。而OCT4表达与远处转移密切相关(*P* < 0.001), 且与转移后化疗疗效明显呈负相关。研究也发现OCT4的表达与肺腺癌患者的疾病进展及预后明显相关, OCT4^+^的患者易发生转移, 病情进展更快, 预后更差, 具有明显的统计学差异。这与CSC容易转移, 且对化疗药物耐药有关。目前对于OCT4作为独立影响肺癌患者生存期的因素, 国内外相关文献较少, 本研究病例数有限, 还需进行大样本研究进一步验证。

CSC的典型特征是能够自我更新和多向分化形成肿瘤内的各类癌细胞。CSC通过自我更新复制自身, 而复制产生的另一个子代细胞经过活跃增殖并逐渐分化, 这个癌细胞(称为短暂放大细胞)是引起肿瘤快速生长的根源, 但其本身缺乏致瘤和转移能力。现有的肿瘤放疗、化疗仅能杀灭短暂放大细胞而不能杀灭癌干细胞, 故疗效有限^[12]^。而本研究中有少数标本OCT4表达阴性, 这可能与标本的保留, 染色方法或表达过弱等因素有关, 也有可能由于部分CSC在某些条件下分化为腺癌细胞, 在此过程中失去了自我更新的能力, 称为分化的腺癌细胞, 因而不表达OCT4, 具体原因还有待进一步研究。

本研究通过多因素分析, 发现转移、OCT4表达及转移后化疗疗效均是独立影响生存期的因素。而OCT4表达的患者多对化疗药物耐药, 预后较差。黄进肃等^[13]^分析肺腺癌干细胞对铂类的药物敏感度发现, 肺腺癌干细胞高表达OCT4, 且肺腺癌干细胞对顺铂和卡铂具有明显耐药性, 可能是化疗后肿瘤复发及预后不良的根源。因此, 杀灭CSC具有重要的临床价值, 而OCT4表达是CSC的重要标志, 因此, OCT4可作为选择性杀灭CSC的一个重要靶标, 从而从源头控制肿瘤进展, 达到提高疗效和改善预后的目的。
